# The impact of the COVID-19 pandemic on physical activity and sedentary behavior during pregnancy: a prospective study

**DOI:** 10.1186/s12884-022-05236-3

**Published:** 2022-12-03

**Authors:** Susan Park, Robert T. Marcotte, John W. Staudenmayer, Scott J. Strath, Patty S. Freedson, Lisa Chasan-Taber

**Affiliations:** 1 Department of Biostatistics & Epidemiology, School of Public Health & Health Sciences, University of Massachusetts, 715 North Pleasant Street, Amherst, MA 01003-9304 USA; 2 Department of Kinesiology, School of Public Health & Health Sciences, University of Massachusetts, Amherst, MA USA; 3 Department of Mathematics and Statistics, College of Natural Sciences, University of Massachusetts, Amherst, MA USA; 4 Department of Kinesiology, University of Wisconsin Milwaukee, Milwaukee, WI USA

**Keywords:** Exercise, Pregnancy, Physical activity assessment, COVID-19, Epidemiology

## Abstract

**Background:**

Prior studies evaluating the impact of the COVID-19 pandemic on pregnancy physical activity (PA) have largely been limited to internet-based surveys not validated for use in pregnancy.

**Methods:**

This study used data from the Pregnancy PA Questionnaire Validation study conducted from 2019–2021. A prospective cohort of 50 pregnant women completed the Pregnancy PA Questionnaire (PPAQ), validated for use in pregnancy, in early, mid, and late pregnancy and wore an ActiGraph GT3X-BT for seven days. COVID-19 impact was defined using a fixed date of onset (March 13, 2020) and a self-reported date. Multivariable linear mixed effects regression models adjusted for age, early pregnancy BMI, gestational age, and parity.

**Results:**

Higher sedentary behavior (14.2 MET-hrs/wk, 95% CI: 2.3, 26.0) and household/caregiving PA (34.4 MET-hrs/wk, 95% CI: 8.5, 60.3 and 25.9 MET-hrs/wk, 95% CI: 0.9, 50.9) and lower locomotion (-8.0 h/wk, 95% CI: -15.7, -0.3) and occupational PA (-34.5 MET-hrs/wk, 95% CI: -61.9, -7.0 and -30.6 MET-hrs/wk, 95% CI: -51.4, -9.8) was observed in middle and late pregnancy, respectively, after COVID-19 vs. before. There was no impact on steps/day or meeting American College of Obstetricians and Gynecologists guidelines.

**Conclusions:**

Proactive approaches for the promotion of pregnancy PA during pandemic-related restrictions are critically needed.

## Introduction

As of 2022, the COVID-19 pandemic has led to more than 634 million confirmed cases and 6.6 million deaths worldwide [1]. In addition to the negative health consequences among those infected by the SARS-CoV-2 coronavirus, lockdowns were instituted across many nations in an effort to impede the spread of the virus. These lockdowns have been shown to adversely impact mental health, diet, sleep, financial stability, and social relationships, resulting in unexpected and sudden shifts in human behavior [2, 3]. For example, studies show that physical activity (PA) decreased throughout the pandemic due to pandemic-related restrictions leading to disruptions in the daily routine (e.g., commute, gym closures, work-from-home, loss of job/hours) for many adults [4].

The rise in physical inactivity and sedentary time has been a consistent public health concern, as it remains one of the leading risk factors for noncommunicable disease mortality and morbidity. Despite the known benefits of obtaining adequate PA, women obtain less PA than men [5]. Commonly cited barriers contributing to the of lack of time for exercise include caretaking and work responsibilities. In addition, during pregnancy, women may experience pregnancy-related changes in physiology and behaviors which pose an additional challenge to maintaining adequate PA levels [6].

Physical inactivity during pregnancy is an urgent public health concern and is implicated in excessive gestational weight gain, gestational diabetes, pre-eclampsia, and preterm birth [7]. The American College of Obstetricians and Gynecologists (ACOG) recommends regular PA during pregnancy due to its positive impact on physical fitness, weight management, prevention of gestational diabetes mellitus (GDM), and psychologic well-being [8]. In terms of the latter, the established beneficial impact of PA on anxiety and depression [2, 9] is particularly critical during psychological stress associated with pandemic lockdown restrictions.

However, few studies have studied the impact of the pandemic on PA levels among pregnant women [10–18] and, with the exception of two studies [11, 17], the majority were internet based surveys administered to convenience samples. All of the studies, with the exception of one [13], used self-reported measures of PA and sedentary behavior which were not validated for use during pregnancy. Finally, the one prospective cohort study used a consumer-grade, as opposed to a research-grade, monitor to measure PA (i.e., Samsung smartwatch) [17]. Studies were largely conducted internationally including Denmark, the UK, Japan, China, Finland, Spain, and the United Arab Emirates, with only one [16] conducted in the US.

Therefore, our aim was to prospectively examine the impact of the COVID-19 pandemic on patterns of PA and sedentary behavior in pregnant women in the US using an objective research-grade measure of PA (ActiGraph) and a self-reported questionnaire validated for use in pregnancy (the Pregnancy PA Questionnaire). Using a combination of PA assessment methods is important as objective measures do not provide information on the context of PA, which is important for public health recommendations. The hypothesis was that pandemic-related restrictions would be associated with lower levels of PA and higher levels of sedentary behavior.

## Methods

### Study population

Participants were part of an observational cohort study designed to use novel validation techniques to re-validate the Pregnancy Physical Activity Questionnaire (PPAQ) (NIH/NICHD 1R21HD094565). Enrollment commenced in March 2019 and follow-up continued until January 2021. Participants (N = 50) were recruited in early pregnancy via flyers at prenatal care centers, health clinics, and advertisements in local papers and using paid Facebook™ advertisements.

Participants were screened at recruitment with a single item rating scale for level of PA [19] to ensure they represented a range of people who regularly undertake a diverse set of activities of various intensities (e.g., low active to high active participants). Women were considered ineligible for the study if they had any of the following characteristics: 1) > 20 weeks gestation, 2) under 16 or over 40 years of age, 3) pregnant with twins or triplets as this is often accompanied by clinically prescribed PA restrictions, 4) musculoskeletal issues that limited ambulation, 5) chronic disease (e.g., diabetes requiring insulin, hypertension or heart disease requiring medication, chronic renal disease, emphysema) or life-threatening illnesses, or 6) lack of a telephone.

This research was performed in accordance with the Declaration of Helsinki and was approved by approved by the Institutional Review Board of the University of Massachusetts, Amherst (reference number: 118–0811). Each participant read and signed a written informed consent.

### Study design

A total of three 7-day assessments were conducted in early, mid, and late pregnancy. Women were recruited in early pregnancy (i.e., 8–20 weeks gestation). The late pregnancy assessment was scheduled 4 to 5 weeks prior to the expected delivery date (i.e., 35–36 weeks gestation) and the middle pregnancy assessment was scheduled halfway between the early and late pregnancy assessment. Participants were given the option of an in-person or virtual assessment. At the beginning of each assessment period, the PPAQ was administered and women were instructed in use of the ActiGraph to be worn for the following 7-days. Participants were instructed on how to complete a wear log, which recorded times that ActiGraph was not worn (e.g., during sleeping or water-based activities). Video recorded demonstrations of how to correctly wear the monitors, as well as links to paper-based materials were provided for all participants on a study website. At the end of each assessment period, the PPAQ was repeated.

The impact of the onset of the pandemic was evaluated cross-sectionally at each of the three assessment timepoints (i.e., early, middle, and late pregnancy). A secondary analysis, limited to women whose enrollment periods spanned the pre and post COVID-19 pandemic onset dates, was also conducted.

### Assessment of COVID-19 impact

The impact of the COVID-19 pandemic was defined in two ways. First, the fixed date of March 13, 2020 was used. This date, which represents the timeframe in which states and businesses considered stay-at-home mandates, was selected to be consistent with prior studies that used a mid-March timeframe [11, 16]. PA before March 13, 2020 was therefore defined as “not impacted by COVID-19” and PA after March 13, 2020 was defined as “impacted by COVID-19.”

Secondly, the self-reported COVID-19 impact date was used. Specifically, at each assessment conducted after mid-March of 2020, women were asked, “Has your lifestyle or daily routine been impacted by the COVID-19 pandemic?” For those that responded ‘yes’, PA before the self-reported COVID-19 impact date was therefore defined as “not impacted by COVID-19” and PA after the self-reported COVID-19 impact date was defined as “impacted by COVID-19.”

### Physical activity assessment

PA was assessed via objective measures (ActiGraph) and self-report (the PPAQ) during the three assessment time periods (i.e., early, middle, and late pregnancy).

#### ActiGraph-GT3X-BT activity monitor

Participants were instructed to wear the ActiGraph GT3X-BT (ActiGraph) on the non-dominant wrist for 7 consecutive days except during sleeping or water-based activities (e.g., showering, swimming). ActiGraph devices were initialized to collect data at 80 Hz using ActiLife 6 software (version 6.13.1: ActiGraph, Pensacola, FL). After each assessment period, data were exported as raw and 1-s epoch files.

Wear time was based on self-report or, if missing, using the Choi wear time algorithm [20]. Only assessment periods with at least 10 h of wear time for at least 4 days were included in analyses [21]. For overall activity metrics, total vector magnitude counts per day and total steps per day were computed using days with valid wear time. For time spent in different behaviors, time and frequency-domain features were computed from raw acceleration data in 15-s non-overlapping epochs and used in previously trained random forest models to classify sedentary and locomotion (walking/running) behaviors [22].

Counts and cut-points were not used to define sedentary behavior and PA because existing cut-point methods are specific to hip-worn ActiGraph accelerometers. In contrast, we used wrist worn accelerometers in our study, as is done in large scale surveillance studies such as NHANES [23] and the UK BioBank [24]. We then used machine learning methods to translate activity patterns from the raw wrist data into more interpretable units (i.e., sedentary behavior and locomotion time) [22].

#### Pregnancy Physical Activity Questionnaire (PPAQ)

The Pregnancy Physical Activity Questionnaire (PPAQ) was used to measure pregnancy PA [25]. The PPAQ is a semi-quantitative instrument that measures the duration and intensity of time spent in household/caregiving, occupational, transportation (i.e., driving and walking to go places), and sports/exercise activities. Intraclass correlation coefficients used to measure reproducibility of the PPAQ were 0.78 for total activity, 0.82 for moderate activity, 0.81 for vigorous activity, and ranged from 0.83 for sports/exercise to 0.93 for occupational activity. Spearman correlations between the PPAQ and 3 published accelerometer cutpoints used to classify ActiGraph data ranged from 0.08 to 0.43 for total activity, 0.25 to 0.34 for vigorous-intensity activity, 0.20 to 0.49 for moderate-intensity activity, -0.08 to 0.22 for light-intensity activity, and -0.34 to 0.12 for sedentary behavior [25]. These validity findings were comparable to those for prior PA questionnaires in non-pregnant populations.

Participants were asked to complete the PPAQ twice at each of the three assessment periods (i.e., early, middle, and late pregnancy). The PPAQ was modified to capture the full range of contemporary sedentary behavior (e.g., ‘screen-time’ via smart phones and tablets; time spent texting and on social media). The number of minutes spent in each reported activity was multiplied by its metabolic equivalent of task (MET) level to estimate average weekly MET-hours/week. MET intensity scores were based on the 2011 Compendium of Physical Activities [26] except for walking and light housework activities for which field-based measures among pregnant women [27, 28] were used. Activities were classified by PA type (e.g., sports/exercise, household/caregiving, occupational, and transportation), and PA intensity (e.g., vigorous, moderate, light, and sedentary). Consistent with the ACOG guidelines [8] as well as the 2019 Canadian Guidelines for physical activity in pregnancy [29], meeting ACOG guidelines was defined as obtaining at least 150 min of moderate-vigorous intensity sports/exercise activity per week (i.e., the equivalent of > 7.5 MET-hrs/week) [8].

### Covariates

Information on age, first day of last menstrual period, expected delivery date, and parity were collected via self-report at the beginning of each assessment period. During the early pregnancy in-person assessment, the researcher measured height and weight using a stadiometer and a calibrated scale, respectively; for virtual assessments this information was collected via self-report. Body mass index was calculated as kg/m^2^. Gestational age (weeks) was calculated using first day of last menstrual period and/or expected delivery date.

### Statistical analysis

Linear mixed effects regression models were used to cross-sectionally evaluate the impact of COVID-19 on PA and sedentary behavior at each of the three assessment timepoints (i.e., early, middle, and late pregnancy). Established risk factors for PA and sedentary behavior in models (i.e., age, early pregnancy BMI, gestational age, parity) were included. Models with ActiGraph PA outcomes were further adjusted for wear time minutes. Chi-square likelihood ratio tests were used to identify whether COVID-19 had a statistically significant impact on PA and sedentary behavior outcomes using an alpha level of 0.05.

A secondary prospective analysis among women whose enrollment period spanned the pre and post COVID-19 pandemic onset dates was then conducted. This analysis, therefore, excluded women whose follow-up ended before the COVID-19 impact date or who were enrolled after the COVID-19 impact date. Sociodemographic and medical history characteristics of this prospective subsample were compared to the total sample using 2-sample t-tests for quantitative characteristics and Chi-square tests for categorical characteristics. Linear mixed effects regression models with random intercept for participants were used to evaluate the impact of COVID-19 on PA and sedentary behavior.

All analyses were conducted using R (version 3.6.1).

## Results

Among the overall sample (*N* = 50), women were on average 32.9 ± 4.2 years, non-Hispanic white (80%), and overweight (25.1 ± 3.6 kg/m^2^) (Table 1) with 90–100% reporting attending school or working during follow-up. Six women had missing PPAQ data at the middle (*n* = 3) and late (*n* = 6) pregnancy assessment time points. One woman was excluded from the ActiGraph analyses for not meeting the criteria of 10 h of wear time for at least 4 days in the early, middle, and late pregnancy assessment periods.

**Table 1:** Characteristics of the Study Sample

Characteristics	Overall Sample
N	50
**ActiGraph Measures**	
Total vector magnitude (1000 counts/day)	2154.7 ± 738.9
Average Steps/day	8334 ± 2999
Sedentary Time	
min/day	516.6 ± 116.6
% time	63.5 ± 11.6
Locomotion (Walking/Running)	
min/day	12.6 ± 13.9
% time	1.6 ± 1.7
**PPAQ Measures (MET-hrs/wk)**	
Meeting ACOG guidelines^a^ (%)	47%
Total physical activity	153 ± 49.5
Type	
Household/Caregiving	61.0 ± 40.6
Sport/Exercise	9.6 ± 8.9
Occupational	45.6 ± 36
Transportation	14.5 ± 12.9
Intensity	
Light	36.3 ± 25.5
Moderate	62.0 ± 34.9
Vigorous	2.3 ± 5.5
Sedentary	52.4 ± 24.3
**Covariates**	
Age (years)	32.9 ± 4.2
Prepregnancy body mass index (kg/m^2^)	25.1 ± 3.6
Parity (prior pregnancies)	1.1 ± 1.4
Mean gestational age (wks)	
Early pregnancy	14.9 ± 3.8
Middle pregnancy	26.0 ± 3.0
Late pregnancy	35.9 ± 1.6
Non-Hispanic White (%)	80%
Wear Time (hrs/day)	13.5 ± 1.6

^a^American College of Obstetricians and Gynecologists of ≥ 7.5 MET hours/week of moderate-vigorous intensity sports and exercise

Firstly, the impact of the onset of the pandemic within each assessment period (i.e., early, mid, and late pregnancy) was evaluated. Mean gestational age was 14.7 ± 3.8 in early, 26.3 ± 3 in middle, and 36 ± 1.3 weeks in late pregnancy.

Using the fixed COVID-19 impact date and PPAQ measures, higher levels of sedentary behavior (14.2 MET-hrs/wk, 95% CI: 2.3, 26.0) in early pregnancy assessments completed after the pandemic as compared to those completed before the pandemic were observed (Table 2). However, the impact of the pandemic on other PPAQ measures (i.e., meeting ACOG guidelines, household/caregiving, sports/exercise, occupational, transportation, light, moderate, or vigorous activity) or ActiGraph measures (i.e., locomotion, steps/day, sedentary time) among women who participated in assessments held prior to or after the fixed COVID-19 impact date was not observed.

**Table 2:** Cross-Sectional Association of COVID-19 on Pregnancy Physical Activity and Sedentary Behavior using a Fixed Impact Date

Variable	Early Pregnancy		Mid Pregnancy		Late Pregnancy	
	β (95% CI)^a^	*p*-value	β (95% CI)^a^	*p*-value	β (95% CI)^a^	*p*-value
ActiGraph Measures						
Total vector magnitude (1000 counts/day)	-91.8 (-458.4, 274.8)	0.62	106.2 (-269.5, 483.4)	0.57	-149.7 (-530.1, 242.9)	0.48
Average Steps/day	-573.0 (-2134.0, 987.6)	0.46	-124.7 (-1623.4, 1378.3)	0.87	-880.0 (-2412.9, 712.7)	0.29
Sedentary Time						
min/day	17.8 (-27.0, 62.6)	0.43	5.1 (-42.9, 53.1)	0.84	16.3 (-33.5, 65.0)	0.54
% time	1.9 (-3.5, 7.4)	0.48	0.6 (-5.5, 6.7)	0.85	1.5 (-4.8, 7.6)	0.66
Locomotion (Walking/Running)						
min/day	-2.2 (-7.8, 3.4)	0.43	-5.0 (-12.3, 2.3)	0.18	-5.4 (-11.3,1.0)	0.1
% time	-0.3 (-1.0, 0.5)	0.48	-0.6 (-1.4, 0.3)	0.21	-0.6 (-1.3, 0.2)	0.14
PPAQ Measures (MET-hrs/wk)						
Meeting ACOG guidelines* (%)	0.1 (-2.1, 2.4)	0.9	1.6 (-2.6, 9.5)	0.4	0.6 (-3.0, 5.0)	0.72
Total physical activity	7.8 (-14.6, 30.0)	0.5	4.1 (-26.4, 34.5)	0.79	18.3 (-13.6, 52.1)	0.24
By type						
Household/Caregiving	-1.4 (-20.7, 18.0)	0.89	23.3 (-0.7, 47.3)	0.06	12.7 (-3.9, 33.3)	0.12
Sport/Exercise	5.2 (-0.5, 10.2)	0.08	-0.5 (-6.3, 5.4)	0.87	0.5 (-3.1, 3.9)	0.9
Occupational	5.3 (-10.5, 20.8)	0.52	-21.9 (-47.3, 3.6)	0.09	-12.5 (-36.1, 10.6)	0.28
Transportation	-4.5 (-11.3, 2.2)	0.18	-8.1 (-14.5, -1.6)	0.02	-2.6 (-9.0, 3.7)	0.41
By intensity						
Light	-4.0 (-19.1, 10.8)	0.57	-6.4 (-21.9, 9.1)	0.41	-0.6 (-11.2, 10.6)	0.95
Moderate	-1.8 (-16.5, 13.0)	0.81	8.2 (-11.2, 27.6)	0.4	21.6 (-1.5, 45.9)	0.07
Vigorous	0.3 (-2.4, 3.1)	0.8	-2.5 (-6.7, 1.7)	0.23	-0.6 (-2.8, 1.4)	0.52
Sedentary	14.2 (2.3, 26.0)	0.02	4.8 (-11.9, 1.5)	0.57	-1.6 (-15.1, 12.2)	0.83

^*^American College of Obstetricians and Gynecologists of ≥ 7.5 MET hours/week of moderate-vigorous intensity sports and exercise

^a^Models adjusted for age, BMI, gestational age, and parity. ActiGraph measures were also adjusted for wear time

Using the self-reported COVID-19 impact date and ActiGraph measures, less locomotion was observed in middle pregnancy assessments (-8.0 h/wk, 95% CI: -15.7, -0.3) held after the pandemic as compared to assessments held before the pandemic in adjusted analyses (Table 3). In terms of PPAQ measures, compared to the pre-pandemic time period, greater household/caregiving activity was observed in middle and late pregnancy assessments held after the pandemic (34.4 MET-hrs/wk, 95% CI 8.5, 60.3 and 25.9 MET-hrs/wk, 95% CI: 0.9, 50.9, respectively) as compared to assessments held before the pandemic. This would be equivalent to, for example, 7–9 h per week in raking/gardening or other moderate intensity household activity. In addition, compared to the pre-pandemic time period, less occupational activity was observed in middle and late pregnancy assessments held after the pandemic (-34.5 MET-hrs/wk, 95% CI: -61.9, -7.0 and -30.6 MET-hrs/wk, 95% CI: -51.4, -9.8). This would be equivalent to, for example, 7–8 fewer hours per week walking quickly at work.

**Table 3:** Cross-Sectional Association of COVID-19 on Pregnancy Physical Activity and Sedentary Behavior using a Self-Reported Impact Date

Variable	Early Pregnancy		Mid Pregnancy		Late Pregnancy	
	β (95% CI)^a^	*p*-value	β (95% CI)^a^	*p*-value	β (95% CI)^a^	*p*-value
ActiGraph Measures						
Total vector magnitude (1000 counts/day)	-159.8 (-599.2, 279.4)	0.47	30.4 (-379.9, 442.1)	0.88	-239.6 (-607.4, 127.9)	0.47
Average Steps/day	-1135.3 (-2991.6, 719.6)	0.22	-723.8 (-2337.9, 894.8)	0.37	-1234.1 (-2695.1, 224.8)	0.22
Sedentary Time						
min/day	35.8 (-17.2, 88.9)	0.18	12.6 (-39.6, 64.6)	0.63	29.0 (-17.8, 75.8)	0.22
% time	4.2 (-2.3, 10.7)	0.20	1.7 (-5.0, 8.3)	0.62	3.0 (-2.9, 8.9)	0.30
Locomotion (Walking/Running)						
min/day	-2.8 (-9.5, 3.9)	0.10	-8.0 (-15.7, -0.3)	0.04	-2.9 (-8.8, 3.0)	0.10
% time	-0.3 (-1.2, 0.5)	0.47	-0.9 (-1.8, 0.0)	0.05	-0.3 (-1.0, 0.4)	0.47
PPAQ Measures (MET-hrs/wk)						
Meeting ACOG guidelines* (%)	-0.2 (-2.9, 2.5)	0.88	3.6 (-0.9, 12.8)	0.12	0.2 (-3.8, 4.2)	0.90
Total physical activity	5.6 (-20.9, 32.0)	0.67	2.7 (-31.4, 36.8)	0.88	-4.9 (-38.8, 29.1)	0.78
By type						
Household/Caregiving	2.6 (-20.9, 26.0)	0.83	34.4 (8.5, 60.3)	0.01	25.9 (0.9, 50.9)	0.04
Sport/Exercise	1.6 (-4.3, 7.5)	0.59	3.4 (-3.1, 9.8)	0.29	0.2 (-4.9, 5.3)	0.93
Occupational	1.3 (-17.8, 20.4)	0.89	-34.5 (-61.9, -7.0)	0.02	-30.6 (-51.4, -9.8)	0.01
Transportation	-0.5 (-8.9, 7.9)	0.90	-5.7 (-13.2, 1.9)	0.14	-4.9 (-11.9, 2.2)	0.17
By intensity						
Light	-8.9 (-27.3, 9.5)	0.33	-3.4 (-20.9, 14.0)	0.69	-1.0 (-16.5, 14.5)	0.90
Moderate	-0.6 (-17.9, 16.8)	0.95	8.1 (-13.7, 29.8)	0.46	2.8 (-21.1, 26.7)	0.81
Vigorous	2.1 (-1.4, 5.7)	0.23	-0.5 (-5.3, 4.3)	0.83	-0.8 (-3.6, 2.0)	0.57
Sedentary	13.0 (-1.4, 27.3)	0.08	-1.5 (-20.2, 17.2)	0.87	-5.9 (-21.2, 9.3)	0.44

^*^American College of Obstetricians and Gynecologists of ≥ 7.5 MET hours/week of moderate-vigorous intensity sports and exercise

^a^Models adjusted for age, BMI, gestational age, and parity. ActiGraph measures were also adjusted for wear time

Other ActiGraph and PPAQ-measured PA outcomes did not significantly differ among women who participated prior to or after the self-reported COVID-19 impact date.

A secondary analysis to evaluate the prospective impact of the COVID-19 pandemic was conducted. Women whose follow-up ended entirely prior to the COVID-19 impact date or whose enrollment started after the COVID-19 impact date were excluded from this prospective analysis leaving a final sample size of *n* = 20. Although women in this subgroup did not significantly differ from the overall study sample in terms of any sociodemographic or medical history characteristics (all *p* > 0.05), it is important to note that the sample size was small and therefore may not reflect true population characteristics.

Compared to the pre-pandemic time period defined by the fixed COVID-19 impact date, locomotion (-6.2 h/week, 95% CI: -11.5, -1.5) and percent of time in locomotion (-0.7%, 95% CI: -1.3, -0.1) as measured by the ActiGraph significantly decreased after the onset of the pandemic adjusting for age, early pregnancy BMI, gestational age, parity, and wear time (Table 4). This would be equivalent to, for example, a decrease of almost 6 h per week in walking to go places. Similarly, using the PPAQ measures, statistically significant decreases in transportation activity (-19.7 MET-hrs/wk, 95% CI:—27.4, -13.7) and increases in household/caregiving activity (19.2 MET-hrs/wk, 95% CI: 1.8, 38.0) were observed after the onset of the pandemic (Table 4) as compared to the pre-pandemic time period. This would be equivalent to, for example, 5 h per week in raking/gardening or other moderate intensity household activity. Similar reductions in transportation activity (-16.7 MET-hrs/wk, 95% CI: -24.7, -8.8) were observed using the self-reported COVID-19 impact date. An impact of the pandemic on other PPAQ measures (i.e., meeting ACOG guidelines, household/caregiving, sports/exercise, occupational, light, moderate, or vigorous intensity activity) was not observed.

**Table 4:** Prospective Impact of COVID-19 on Pregnancy Physical Activity and Sedentary Behavior

**Variable**	**Fixed COVID-19 Date**		**Self-Reported COVID-19 Date**	
	β (95% CI)^a^	*p*-value	β (95% CI)^a^	*p*-value
ActiGraph Measures				
Total vector magnitude (1000 counts/day)	-36.3 (-285.8, 225.8)	0.82	10.1 (-268.1, 284.5)	0.95
Average Steps/day	-214.0 (-1221.0, 794.7)	0.68	-83.8 (-1226.5, 967.5)	0.82
Sedentary Time				
min/day	8.8 (-25.2, 42.5)	0.61	5.9 (-30.1, 43.3)	0.72
% time	1.2 (-3.0, 5.4)	0.57	0.6 (-3.8, 5.3)	0.75
Locomotion (Walking/Running)				
min/day	-6.2 (-11.5, -1.5)	0.01	-3.3 (-9.6, 1.6)	0.35
% time	-0.7 (-1.3, -0.1)	0.02	-0.3 (-1.1, 0.3)	0.29
PPAQ Measures				
Meeting ACOG guidelines^b^ (%)	0.8 (-1.3, 2.9)	0.45	0.4 (-2.1, 2.7)	0.74
Total physical activity	3.4 (-20.8, 28.4)	0.76	-3.1 (-29.2, 26.3)	0.91
By type				
Household/Caregiving	19.2 (1.8, 38.0)	0.03	9.6 (-10.0, 31.6)	0.31
Sport/Exercise	1.2 (-3.9, 6.5)	0.62	-0.3 (-5.7, 6.2)	0.95
Occupational	-7.4 (-22.1, 5.2)	0.22	-4.5 (-20.7, 9.7)	0.48
Transportation	-19.7 (-27.4, -13.7)	< 0.001	-16.7 (-24.7, -8.8)	< 0.001
By intensity				
Light	-5.9 (-16.4, 4.4)	0.25	-4.9 (-16.3, 7.0)	0.43
Moderate	11.1 (-6.9, 30.0)	0.22	1.8 (-17.8, 24.4)	0.77
Vigorous	-2.2 (-6.2, 1.7)	0.25	-2.1 (-6.4, 2.5)	0.39
Sedentary	0.5 (-10.0, 11.4)	0.90	3.4 (-8.4, 15.4)	0.56

^a^Models adjusted for age, BMI, gestational age, and parity. ActiGraph measures were also adjusted for wear time

^b^American College of Obstetricians and Gynecologists of ≥ 7.5 MET hours/week of moderate-vigorous intensity sports and exercise

## Discussion

In this analysis of the impact of the COVID-19 pandemic on PA during pregnancy using both objective and self-reported measures of PA and sedentary behavior, it was found that locomotion and transportation PA decreased, and household/caregiving PA increased after the onset of the COVID-19 pandemic using the fixed COVID-19 impact date of March 13, 2020. Additionally, higher levels of sedentary behavior in early pregnancy assessments held after the fixed COVID-19 impact date as compared to assessments held before the pandemic were observed. No impact of the pandemic on steps/day, total vector magnitude, occupational, sports/exercise activity, or meeting ACOG guidelines.

Consistent with our findings, the majority of the prior cross-sectional studies found that the COVID-19 pandemic negatively impacted PA during pregnancy [10–12, 15, 18]. For instance, Hillyard et al. conducted an internet based survey among 724 pregnant participants with gestational diabetes mellitus in the UK [12]. Using a single-item PA measure, the authors found that while almost half (47%) reported meeting PA guidelines pre COVID-19, this dropped to 23% during the COVID-19 pandemic while sedentary time increased for 79% of participants. In contrast, in the only study to use a validated measure of pregnancy PA and sedentary behavior (i.e., the Japanese PPAQ), Hori et al. conducted an internet based survey among 168 pregnant women in their second and third trimesters in Japan [13]. The authors found no significant differences in PA (i.e., total PA, intensity and type of activity, and inactivity) between women who reported being affected by the pandemic as compared to those who were unaffected.

Our prospective findings are similar to the one prior prospective study that examined PA patterns in pregnant women before and during the COVID-19 pandemic. In this study among 38 Finnish pregnant women [17], the authors found that device-estimated step counts, using a Samsung smartwatch, decreased (*p* = 0.001) and inactive time increased (*p* = 0.014) after the COVID-19 national restrictions. Although there was no impact of the COVID-19 pandemic on steps or sedentary behavior in our prospective analyses, a decrease in locomotion and transportation PA was observed.

Differences in findings between our study and prior studies may have occurred due to the differences in the tools used to measure PA. For example, prior research has shown large variability between step estimates from research-grade (ActiGraph) and consumer-grade (e.g., Samsung, Fitbit) devices [30, 31]. For self-reported measures, the detailed Pregnancy Physical Activity Questionnaire was used, as did one prior cross-sectional study by Hori et al. [13] In contrast, the one prior prospective study relied upon a single-item Likert-scale PA (0–100) question. Lastly, studies varied in their measures of COVID-19 impact with the majority relying upon the self-reported impact of COVID-19.

Our study had several strengths including the use of a research grade device (ActiGraph) and a questionnaire validated for use in pregnancy to measure PA according to multiple domains of type and intensity. The use of both a fixed COVID-19 impact date as well as a self-reported impact date) to take into account individual-level variability in how women perceived being affected by the COVID-19 pandemic were also study strengths. Finally, our secondary prospective design allowed us to assess the temporal association of the COVID-19 pandemic on PA during pregnancy, although the sample size for this analysis was small and findings should be interpreted with caution.

Our study also had several limitations. First, self-reported information on PA is subject to error. A recent systematic review of physical activity questionnaires for pregnancy recommended the PPAQ to assess PA in pregnancy concluding that it has sufficient reliability in assessing total physical activity and vigorous physical activity. However, the authors noted that the PPAQ revealed insufficient construct validity in assessing these scores, but that the evidence for this was of low-to moderate quality [32]. The review article, therefore, called for future research on the validity of comparison instruments in pregnancy followed by consensus on validation reference criteria. Such error, if it occurred, would tend to bias our results toward the null.

On a related note, while the PPAQ asked participants if they attended school or work, either for wages or as a volunteer during pregnancy, we did not have more detail on the percent working from home nor regarding which careers could be accommodated via work at home during the pandemic.

Secondly, the use of a fixed date (March 13, 2020) to define the onset of COVID-19 may not be individually applicable to all participants. However, the similar associations in our findings using either the fixed or self-reported COVID-19 impact date suggests that this source of bias may be minimal. Third, PA tends to decrease as the pregnancy progresses [33], and our results, and those of prior studies, may partly be explained by normal changes during pregnancy rather than by a change due to the COVID-19 pandemic. However, the consistency of our cross-sectional findings conducted within each stage of pregnancy (early, mid, and late) with our prospective subanalysis, although limited by a small sample size, help to reduce this concern. Lastly, our participants were largely non-Hispanic white women. Therefore, findings may not be generalizable to all pregnant women in the US. For example, studies show that factors related to the COVID-19 pandemic such as employment loss during the onset of the pandemic may differentially affect people of color [34].

In summary, the current study adds to the growing body of research on the impact of the COVID-19 pandemic on PA and sedentary behavior during pregnancy. Locomotion, transportation, occupational PA, and sedentary behavior were negatively impacted by the COVID-19 pandemic and that household/caregiving activity increased. Reduced levels of pregnancy PA are of concern as this may increase the risk of pregnancy complications such as preeclampsia, gestational diabetes mellitus, and increased gestational weight gain. Furthermore, the mental health impact of a pandemic and its associated restrictions will likely be further exacerbated by increases in stress and anxiety which have been associated with low levels of PA [2]. Future studies should investigate whether such negative lifestyle changes persist over time.

Our findings also have implications for the promotion of PA during pandemic-related restrictions. For example, when pandemic lockdowns are initiated, prenatal health care providers should be instructed to address barriers and facilitators to PA and sedentary behavior in their counseling sessions. Resources to help pregnant women maintain healthy lifestyles during pandemics should be tailored for specific racial/ethnic subgroups and take into account diversity in socioeconomic status and access to resources. These may include increased accessibility of online PA classes, provided by instructors qualified in prenatal exercise, as well as resources on how to be active at home, with limited space and equipment.

## Data Availability

The datasets analyzed during the current study are available from the corresponding author on reasonable request.

## Abbreviations

MET: Metabolic equivalent of task
CI: Confidence interval
Hrs: Hours
Wk: Week
PA: Physical activity
ACOG: American College of Obstetricians and Gynecologists
GDM: Gestational diabetes mellitus
